# HIV Testing and Tolerance to Gender Based Violence: A Cross-Sectional Study in Zambia

**DOI:** 10.1371/journal.pone.0071922

**Published:** 2013-08-21

**Authors:** Sara Gari, Jacob R. S. Malungo, Adriane Martin-Hilber, Maurice Musheke, Christian Schindler, Sonja Merten

**Affiliations:** 1 Department of Epidemiology and Public Health, Swiss Tropical and Public Health Institute, University of Basel, Basel, Switzerland; 2 Department of Population Studies, University of Zambia, Lusaka, Zambia; 3 Swiss Centre of International Health, Swiss Tropical and Public Health Institute, University of Basel, Basel, Switzerland; Vanderbilt University, United States of America

## Abstract

This paper explores the effect of social relations and gender-based conflicts on the uptake of HIV testing in the South and Central provinces of Zambia. We conducted a community-based cross-sectional study of 1716 randomly selected individuals. Associations were examined using mixed-effect multivariable logistic regression. A total of 264 men (64%) and 268 women (56%) had never tested for HIV. The strongest determinants for not being tested were disruptive couple relationships (OR = 2.48 95% CI = 1.00–6.19); tolerance to gender-based violence (OR = 2.10 95% CI = 1.05–4.32) and fear of social rejection (OR = 1.48 95% CI = 1.23–1.80). In the Zambian context, unequal power relationships within the couple and the community seem to play a pivotal role in the decision to test which until now have been largely underestimated. Policies, programs and interventions to rapidly increase HIV testing need to urgently address gender-power inequity in relationships and prevent gender-based violence to reduce the negative impact on the lives of couples and families.

## Introduction

Gender inequity is intimately linked to HIV/AIDS. Without addressing gender inequity AIDS will remain a substantial problem. There is evidence showing that gender-power inequity in relationships and gender-based violence (GBV) increases vulnerability to HIV infection [1]–[4]. In Zambia, the HIV prevalence among young women aged 15–24 is more than twice that of men in the same age category [5]–[6]. A number of factors resulting from gender inequity contribute to this higher prevalence. In Zambia, women have practically no ability to refuse sex or to demand the use of condom, a demonstration of their limited agency in sexual relationships. Age-mixing sexual patterns between young girls and older men also play an important role on their greater susceptibility to HIV. [7], [8].

Recent strategies to improve testing rates in Zambia have included the strengthening of provider-initiated HIV testing and counseling [9], and home-based HIV counseling and testing [HBCT] [10], [11]. These strategies however have not yet achieved a sufficient increase in the uptake of testing [12]. In 2009 only 23% of the Zambian population voluntarily requested an HIV test and this percentage was slightly higher among women (25%) than men (21%) [12]. Although this figure indicated notable progress (7% in 2005), the overall testing rate remains low. Common barriers to HIV testing are low levels of education [13]–[16], accessibility issues [13, 14 17–19], concerns about confidentiality and privacy [9], [10], [20], [21], discrimination from health workers and stigmatizing attitudes towards HIV/AIDS in the community [22]–[24]. Recent studies have shown that fear of being rejected by family or abandoned by one’s partner is an important reason why people delay or refuse HIV testing [25]. There is evidence showing that a positive HIV diagnosis can lead to a variety of negative effects such as gender-based violence and loss of social and family support [26], [27].

While most studies on gender inequity and AIDS have focused on examining the relationship between vulnerability to HIV and gender-based violence [27], [28], few have considered the effect on access to HIV care. Some recent studies from Zambia show that women who have suffered gender-based violence often are more likely to choose not to receive treatment because they are afraid of violent behavior and abandonment by family [29], [30]. These studies are based on clinical practice and focused on women who were victims of gender-based violence. There is no published research of large community based studies that examine the relationship between social relationships, tolerance of gender-based violence and HIV testing. We hypothesized that the relational dynamics of one’s social relationships; the level of tolerance of gender-based violence and the fear of such abuses determine decisions about HIV testing. This study tested this hypothesis using social cohesion indicators measured at three different socio-relational levels: the couple, the family and the neighborhood.

## Methods

### Ethical Statement

This study was approved by the EKBB Ethical Committee (Ethik-Kommission beider Basel) and by the Humanities and Social Sciences Research Ethics Committee of the University of Zambia. Clearance was obtained from the Zambian Ministry of Health.

We conducted a community based cross-sectional study of 1716 randomly selected individuals in South and central provinces of Zambia (Chivuna, Mbeza, Mazabuka and Lusaka). We used three-stage sampling design (Primary sampling units (PSUs)-Households-Individuals) to derive our sample. First, we randomly selected PSU’s (chiefdoms or municipalities) in the four study areas from a list of enumeration areas obtained from the last census conducted in Zambia in 2010. We calculated the number of households using a probability proportional to size (PPS) sampling strategy. Households were randomly selected from household lists with the exception of Lusaka where a random walk scheme was used. In each household our interviewers selected one permanent resident >18 years using the Kish within-household respondent selection method [31]. With this method we ensured equiprobability of selection among individuals who fall within the scope of the survey (thus every eligible individual in the household – in our case, all those aged 18 and over – had the same chance of being selected). If the selected individual was not present, an appointment was made. Only if this appointment was missed, a new household was selected. In Lusaka we additionally applied a weighted sampling strategy to build a representative sample of the population living in high, medium and low density areas. A team of 30 experienced Zambian surveyors, who were trained in survey techniques and sexual and reproductive health (SRH) issues, conducted the interviews in the respondents' mother tongue. They obtained signed informed consents from the participants prior to start the interview. Interviews were done between September 2010 and February 2011. Participants who requested it were offered psychosocial counseling by qualified professionals and obtained referral information for local HIV/AIDS voluntary counseling and testing (VCT) services.

The questionnaire included questions on demographic and socioeconomic characteristics, food insecurity, health risk behaviors, social cohesion, anticipated stigma and fear of social rejection and HIV/AIDS-related beliefs. The selection of these themes was guided by the results of an ethnographic study of barriers to HIV treatment conducted in the same areas over the previous year. Additionally we systematically reviewed the literature to identify instruments and scales used by other studies to measure similar themes. A panel of national and international experts reviewed these instruments and scales and selected pertinent questions and items to measure each theme. When necessary we reformulated and adapted questions and items to the Zambian context. We originally created the questionnaire in English and translated it into Nyanja, Ila and Tonga. It was pilot tested twice before final validation. To ensure the confidentiality of the participants’ information we anonymised the questionnaires using numerical codes.

### Measures

***Socio-demographic questions*** were adapted from the Zambian Sexual Behavior Survey [32].

***Cohesion of social relationships*** was assessed at three levels. We adapted partner and family relationships items from the Family Assessment Device (FAD) [33]. We asked the respondents to score their agreement on eight statements (items) using a five point visual scale that we previously piloted on 50 respondents with similar characteristics. Participants with higher scores were considered to have more social (dis)-cohesion and vice versa. To evaluate whether the eight items formed a one-dimensional homogenous scale we performed Mokken Scale analysis for polytomous items [34], using the STATA 12.1 command MSP [35]. Mokken models belong to the class of statistical models called non-parametric item response theory (NIRT). The crucial aspect of the Mokken scale analysis is its ability to establish hierarchies of items ordered by ‘difficulty’ (facility) such that any individual who endorses a particular item should endorse one with a lower difficulty. Mokken scales require three basic assumptions: (1) unidimensionality (one latent variable summarizes the variation in the item score in the questionnaire), (2) local independence (item score are statistically independent conditional on the value of the latent trait), and (3) monotonicity (for all items the probability of a positive response increases monotonically with increasing values of the latent trait). Scale homogeneity is based on Loevinger’s index of homogeneity H [36]. As a rule of thumb Loevinger’s coefficient H <0.30 indicates poor scalability properties, for 0.30< H <0.40 the scale is weak; for 0.40< H <0.50 the scale is medium, and for H >0.50 the scale is strong. The reliability of Mokken scales is estimated using Rho which is a test-retest reliability coefficient with Rho >0.7 considered to indicate a reliable scale [37]. The items that satisfied the three assumptions of the Mokken analysis can be added up and individual scores are then computed as the rank of the highest endorsed item in this hierarchy, i.e. it is a simple total score (sum of positive responses). This total score is used as an estimate of the level of the latent construct, in our case relationships’ cohesion in each subject. Of the initial eight statements Mokken analysis generated three scales. The first one contained four items measuring couple (dis)-cohesion: *In times of crises I cannot turn to my spouse for support; my partner and I do not get along well; I do not trust my partner; I do not feel supported by my spouse/partner* with H = 0.43. Reliability as measured by Rho = 0.96. A second scale with two items measuring family (dis)-cohesion: *people in this household only help you if they can get something out of it; people in this household do not get along well* with H = 0·43 and Rho = 0.98. A third scale was also created with also two items measuring individual perception to the use of domestic violence in their households: *If someone in the household misuses money it is acceptable to beat him/her; In my household if a wife comes home late without the permission of the husband she will be beaten* with H = 0.40 and Rho = 0.73. Thus, a family and a couple (dis)-cohesion as well as a perceived tolerance to violence scale score were computed as the averaged sum of valid answers 1 to 5. Neighborhood (dis)-cohesion was assessed with two items adapted from the work of Sampson et al. [38]. A neighborhood cohesion scaled score with two items was built using the same method: *People in this neighborhood don’t get along well with each other; People around here are not willing to help their neighbors* with H = 0.40 and Rho = 0.73.

***Perception and beliefs about antiretrovirals (ARVs) and traditional medicine*** items were adapted from previous validated questionnaires used in similar contexts [39], [40]. Three separated scales were defined using MSP. The first included five items on knowledge about ARVs: *ARVs can make me sick; ARVs are not good for children; ARVs can make me impotent; ARVs can kill* (H = 0.36, Rho = 0.99), the second contained four items about traditional medicines: *TM can cure HIV/AIDS; TM are easier to take; TM are easier to access* (H = 0.53, Rho = 0.98); And the third contained two items measuring conspiracy beliefs: *HIV/AIDS was released to eradicate the black race; People who take ARVs are guinea pigs for the government and other organizations* (H = 0.59 and Rho = 0.97). Three scaled scores were computed as the averaged sum of valid answers 1 to 5.

***Health risk behaviours*** were measured with two items assessing alcohol use and sexual risk behavior. Both items were included as individual variables in the regression models.

***Stigma and discrimination*** were conceptualized according to the instrumental-symbolic framework [41]-[42]. Respondents’ experiences of internal and enacted stigma were investigated. Internal stigma integrated indicators of anticipated (expected) and self-stigma (internalized). For this paper only anticipated stigma indicators have been analyzed. Self-stigma and enacted stigma are necessarily linked to a positive test result thus they are outside of the scope of this work. Anticipated stigma was measured using 4 items collected from previous validated scales [43], [44] and adapted to the Zambian context: *People with HIV fully participate in the social events in this community; People infected with HIV loose respect in this community; HIV positive children are bullied by other children in this community; People here believe that children should not play with infected children*. Response categories ranged from 1 (strongly disagree) to 5 (strongly agree). Using MSP command a scale with the three last items was defined (H = 0.41 and Rho = 0.97). A scaled score was computed as the averaged sum of valid answers, 1 to 5.

***Fear of social rejection*** items were adapted from previously validated questionnaires used in similar contexts [39], [40]. Two scales were defined using the same method. One included five items expressing fear of social rejection: *Fear of divorce; Fear of losing friends; Fear of damaging the family reputation; Fear of not being able to get married; Fear of being rejected by sexual partners* (H = 0.74, Rho = 0.99). The second scale included three items about self-efficacy: *Fear of having to take medication forever; Fear of side effects; Fear of not being able to handle a life as an HIV positive person* (H = 0.71, Rho = 0.98). Two scaled scores were computed as the averaged sum of valid answers, 1 to 5.

***Fear of community gossip*** was assessed with a 5 point Likert scale (1 = very afraid of community gossip; 5 = not afraid of community gossip).

***Household food insecurity*** was assessed with a shortened version of the 10-item Radimer/Cornell hunger scale [45]. A scaled score with four items was created with MSP: I worry whether *my food will run out before get money to buy more; I eat less than I think I should because I don’t have enough money for food; I know my child(ren) is/are hungry sometimes, but just can’t afford more food; I can’t feed my child(ren) with a balanced meal because I can’t afford it* (H = 0.65 and Rho = 0.97).

### Data Analysis

We used Stata 12.1 software to perform the statistical analysis. First we ran univariable logistic regression models to assess associations between uptake of HIV testing and each individual item related to couple, family, and neighbor relationships and anticipated stigma. We repeated this analysis using the scored scales. Other associations assessed included health risk behaviors, beliefs related to ARVs and traditional medicines (individual items and score), socio-demographic and socio-economic variables. We also tested interactions with sex. Our initial multivariable mixed model included all variables with p<0.2 and interaction terms with p<0.1 in the univariable analysis. In this paper we wanted to focus on individual factors influencing the decision of undergoing HIV-testing. Therefore we chose to model influences at the primary sampling unit level by random effects. The initial multivariable model was then reduced using backward selection. We dropped the least significant variables, as long as they were not significant according to our chosen critical level. In our case p<0.2 for variables and p<0.1 for interactions.We continued by successively re-fitting reduced models and applying the same rule until all remaining variables were statistically significant.

We also carried out sensitivity checks through subgroup analyses that excluded HIV positive respondents who disclosed their status and respondents who reported a change in the relationship with the partner after being tested.

## Results

The surveyors visited a total of 1750 households in which eligible individuals were randomly identified. A total of 1716 participants (98.6%) responded to the interview questions. In Table 1–2 we summarized the characteristics and proportions of persons ever tested for HIV. A total of 532 (31%) respondents had never been tested. Of those 264 (49.6%) were men and 268 (50.5%) women. Most people tested did it only once (27%) or twice (23%). Half of them (52%) were tested less than 6 months before the survey. Surprisingly, more people living in cities (39%) reported to not have been tested as compared to rural areas (27%). Unmarried people and those who perceived their households poorer than other households in the community were also less likely to be tested.

**Table 1:** Descriptive characteristics of respondents by gender and testing status.

	Men						Women				
	Ever tested		Never tested			Total	Ever tested	Never tested			Total
	444 [35.8]		264[64.2]	p		708	740 [45.1]	268 [54.9]	p		1008
**Age [years]**											
18–24		60 [8.5]	67 [9.5]	*	127 [17.9]		149 [14.8]	63 [6.3]	*	212 [21.0]	
25–34		125 [17.7]	77 [10.9]	*	202 [28.5]		250 [24.8]	57 [5.7]	*	307 [30.5]	
35–44		150 [21.2]	55 [7.8]	*	205 [29.0]		184 [18.3]	56 [5.6]	*	240 [23.8]	
45–54		60 [8.5]	25 [3.5]	*	85 [12.0]		82 [8.1]	36 [3.6]	*	118 [11.7]	
>55		46 [6.5]	36 [5.1]	*	82 [11.6]		69 [6.8]	55 [5.5]	*	124 [12.3]	
**Education**											
None		15 [2.1]	15 [2.1]	*	30 [4.2]		49 [4.9]	20 [2.0]		69 [6.8]	
Primary		210 [29.7]	90 [12.7]	*	300 [42.4]		374 [37.1]	109 [10.8]		483 [47.9]	
Secondary		172 [24.3]	117 [16.5]	*	289 [40.8]		254 [25.2]	109 [10.8]		363 [36.0]	
Tertiary		36 [5.1]	34 [4.8]	*	70 [9.9]		56 [5.6]	21 [2.1]		77 [7.6]	
**Marital status**											
Widowed		10 [1.4]	12 [1.7]	*	22 [3.1]		78 [7.7]	44 [4.4]	*	122 [12.1]	
Married		327 [46.2]	138 [19.5]	*	465 [65.7]		474 [47.0]	103 [10.2]	*	577 [57.2]	
Polygamy		87 [12.3]	27 [3.8]		114 [16.1]		114 [11.3]	13 [1.3]	*	127 [12.6]	
Monogamy		239 [33.8]	109 [15.4]		348 [49.2]		347 [34.4]	88 [8.7]	*	435 [43.2]	
Single		85 [12.0]	96 [13.6]	*	181 [25.6]		134 [13.3]	93 [9.2]	*	227 [22.5]	
Separated/divorced		12 [1.7]	10 [1.4]	*	22 [3.1]		38 [3.8]	23 [2.3]	*	61 [6.1]	
**SES**											
Self-perception of wealth - Poor		297 [41.9]	177 [25.0]		474 [66.9]		504 [50.0]	176 [17.5]		680 [67.5]	
Self-perception of wealth - Rich		132 [18.6]	76 [10.7]		208 [29.4]		197 [19.5]	74 [7.3]		271 [26.9]	
Employed		119 [16.8]	73 [10.3]		192 [27.1]		126 [12.5]	65 [6.4]	*	191 [18.9]	
**Uran/Rural**											
Urban – low/medium density		82 [11.6]	71 [10.0]	*	153 [21.6]		173 [17.2]	84 [8.3]	*	257 [25.5]	
Urban - high density		68 [9.6]	80 [11.3]	*	148 [20.9]		153 [15.2]	91 [9.0]	*	244 [24.2]	
Rural		293 [41.4]	113 [16]	*	406 [57.3]		413 [41.0]	93 [9.2]	*	506 [50.2]	

* p<0.05.

**Table 2:** Testing characteristics of respondents by gender and testing status.

	Men	Women
**Number of HIV tests**		
One	142 [32.0]	172 [23.2]
Two	105 [23.6]	168 [22.7]
Three	52 [11.7]	119 [16.1]
Four or more	66 [14.9]	126 [17.0]
**Time since last test**		
Less than 6 months	236 [53.2]	372 [50.3]
6 to 12 months	109 [24.5]	156 [21.1]
1–2 years	26 [5.9]	63 [8.5]
2–3 years	24 [5.4]	51 [6.9]
More than 3 years	15 [3.4]	33 [4.5]

In Table 3 we displayed the top 10 reasons for not testing. The reasons most often reported were: “*Fear that people gossip about me*”(37%), “*Fear that I’d be rejected by sexual partners*” (36%); “*No-one would marry me*” (35%) and “*Fear that my family’s reputation would be damaged*” (32%). Other reasons were related to perceptions of self-efficacy: “*I would not be able to handle life as HIV positive person*” (29%) and “*I’m afraid to take medication forever*” (29%). Other reasons related to social support were “*I’m afraid of being abandoned by my partner*” (26%) and “*I’m afraid to lose my friends*” (25%). Only one reason was directly related to treatment with ARVs: “*I’m worried about side effects*” (19%).

**Table 3:** Top 10 reasons for non-uptake of HIV testing among non-tested participants.

	N	%
Fear of gossip and finger point in the community	199/532	37%
Fear of being rejected by sexual partners to have sexual intercourse	194/532	36%
Fear of being rejected by potential partners to get married	188/532	35%
Fear of damaging the family reputation	168/532	32%
Fear of not being able to handle a life as an HIV positive person	153/532	29%
Fear of taking medication forever	153/532	29%
Fear of losing the main partner	140/532	26%
Fear of losing friends	135/532	25%
Fear of ARVs’ side effects	103/532	19%
Fear of not being able to have children	100/532	19%

In Tables 4–6 we show the crude odds ratios associated with non-uptake of HIV testing. Living in urban areas (OR = 2.40 95% CI = 1.94–2.96), and being educated (OR = 1.12 95% CI = 1.05–1.21) increased the odds of not being tested while being female (OR = 0.61 95% CI = 0.50–0.75), married (OR = 0.40 95% CI = 0.32–0.49), very religious (OR = 0.83 95% CI = 0.73–0.94) and involved in community activities (OR = 0.55 95% CI = 0.45–0.68) were positively associated with testing. Limited knowledge of ARVs (OR = 1.62 95% CI = 1.09–2.41) and reliance on traditional medicines (OR = 1.29 95% CI = 1.10–1.51) increased the odds of not being tested. Being unemployed (OR = 1.34 95% CI = 1.06–1.71), not owning household assets (OR = 1.74 95% CI = 1.41–2.14), having no power to decide over household resources (OR = 1.31 95% CI = 1.13–1.52) and food insecurity (OR = 1.17 95% CI = 1.02–1.33) increased the risk of non-uptake of testing. Being unaware of where to go for testing was the greatest risk factor (OR = 39.11 95% CI = 14.14–108.20) for not testing but only about 4% of the respondents said they did not know where to go.

**Table 4:** Crude odds ratios for socio-demographic and socio-economic factors.

	N [%]	OR	P value	95% CI	
**Socio-demographic factors**					
Age*		1.01	0.060	1.00	1.01
Urban*	782 [45.6]	2.40	**0.000**	1.94	2.96
Women*	1008 [58.7]	0.61	**0.000**	0.50	0.75
**Education level**					
None *vs*. any education	99 [5.8]	1.25	0.299	0.82	1.92
Primary education	783 [45.6]	0.62	**0.036**	0.40	0.97
Secondary education*	652 [38.0]	0.97	0.893	0.62	1.51
Tertiary education*	147 [8.6]	1.09	**0.004**	0.64	1.85
Religious feeling [the more]*	1395 [81.3]	0.83	**0.004**	0.73	0.94
Do not attend religious services*	394 [23.0]	1.25	**0.000**	1.10	1.41
Participates in community activities*	864 [50.3]	0.55	**0.000**	0.45	0.68
Married*	1042 [60.7]	0.40	**0.000**	0.32	0.49
Widow or divorce	227 [13.2]	1.53	**0.004**	1.15	2.04
**Socio-economic factors**	383 [22.3]				
Employment [any]*	741 [43.2]	1.34	**0.016**	1.06	1.71
Does not own any household assets*	201 [11.7]	1.74	**0.000**	1.41	2.14
Cannot decide on household resources*	309 [18.0]	1.31	**0.000**	1.13	1.52
Often ate less than wanted due to lack of money to buy food*	782 [45.6]	1.17	**0.020**	1.02	1.33

* statistical significance p<0.05.

**Table 5:** Crude odds ratios beliefs about HIV and ARVs.

	N [%]	OR	P value	95% CI	
Does not know any place to go for testing*	65 [3.8]	39.11	**0.000**	14.14	108.20
ARVs can make sick*	246 [14.3]	1.34	**0.050**	1.00	1.80
ARVs are not good for children*	276 [16.1]	1.39	**0.023**	1.05	1.84
ARVs can make impotent	95 [5.5]	1.39	0.143	0.89	2.16
ARVs can kill	78 [4.5]	1.07	0.654	0.80	1.43
Health literacy [ARVs] score* 1	131 [7.6]	1.62	**0.017**	1.09	2.41
Traditional medicine [TM] can cure HIV/AIDS#	36 [2.1]	0.97	0.698	0.83	1.14
TM are easier to take	81 [4.7]	1.09	0.195	0.96	1.23
TM are easier to access	89 [5.2]	1.09	0.169	0.96	1.23
TM belief score* 2	108 [6.3]	1.29	**0.002**	1.10	1.51
HIV can be caused by witchcraft	1405 [81.9]	1.10	0.112	0.98	1.24
HIV/AIDS was release to eradicate the black race	124 [7.2]	1.11	0.056	1.00	1.23
People who take ARVs are guinea pigs for the government	122 [7.1]	1.08	0.201	0.96	1.20
Conspiracy beliefs score^3^	124 [7.2]	1.11	0.074	0.99	1.24

* statistical significance p<0.05.

# reported per one unit increase in scale 1 to 5.

1 score included:ARVs can make sick; ARVs are not good for children; ARVs can make impotent; ARVs can kill.

2 score included: TM can cure HIV/AIDS; TM are easier to take; TM are easier to access.

3 score included: HIV/AIDS was release to eradicate the black race; People who take ARVs are guinea pigs for the government and other organizations.

**Table 6:** Crude odds ratios of social support factors.

	N [%]	OR	P value	95% CI	
**Social support**					
Lack of household support*	113[6.59]	0.79	**0.000**	0.70	0.89
Lack of support from partner*	1027 [59.8]	1.14	**0.019**	1.02	1.27
Does not get along well with partner/spouses*	1065 [62.1]	1.19	**0.005**	1.06	1.35
Tolerance of interpersonal violence	1346 [78.4]	0.95	0.318	0.86	1.05
Tolerance of gender based violence	208 [12.1]	0.98	0.626	0.89	1.08
**Domestic violence score** 1	554 [32.3]	0.92	0.146	0.83	1.03
People in this neighborhood don’t get along well with each other	255 [14.9]	0.98	0.699	0.89	1.08
People around here are not willing to help their neighbors*	275 [16.0]	1.28	**0.000**	1.17	1.40
**Community cohesion score** * 2	242 [14.1]	1.18	**0.002**	1.06	1.32
Fear of divorce*	428 [24.9]	1.13	**0.001**	1.05	1.21
Fear of losing friends*	375 [21.9]	1.13	**0.001**	1.05	1.21
Fear of damaging the family reputation*	412 [24.0]	1.23	**0.000**	1.14	1.31
Fear of not being able to get married*	467 [27.2]	1.23	**0.000**	1.15	1.31
Fear of being rejected by sexual partners*	496 [28.9]	1.22	**0.000**	1.14	1.31
**Fear of social rejection score** * 3	454 [26.5]	1.25	**0.000**	1.16	1.35
Fear of having to take medication forever*	467 [27.2]	1.08	**0.023**	1.01	1.16
Fear of side effects	342 [19.9]	1.03	0.467	0.95	1.11
Fear of not being able to handle a life as an HIV positive person	478 [27.9]	1.06	0.082	0.99	1.14
**Self-efficacy score** 4	394 [23.0]	1.06	0.148	0.98	1.16
People with HIV loose respect in this community*	405 [23.6]	1.18	**0.002**	1.06	1.31
HIV positive children are bullied by other children in this community	182 [10.6]	1.02	0.777	0.91	1.13
People here believe that children should not play with children infected	154 [9.0]	1.09	0.099	0.98	1.22
**Stigma score** * 5	550 [32.1]	1.21	**0.001**	1.09	1.35
**Fear of community gossip [social control]** *	259 [15.1]	1.12	**0.001**	1.05	1.19

* statistical significance p<0.05.

1 score included: If someone in the household misuses money it is acceptable to beat him/her; In my household if a wife comes home late without the permission of the husband she will be beaten.

2 score included: People in this neighbourhood don’t get along well with each other; People around here are not willing to help their neighbours.

3 score included: Fear of divorce; Fear of losing friends; Fear of damaging the family reputation; Fear of not being able to get married; Fear of being rejected by sexual partners.

4 score included: Fear of having to take medication forever; Fear of side effects; Fear of not being able to handle a life as an HIV positive person.

5 score included: People infected with HIV loose respect in this community; HIV positive children are bullied by other children in this community; People here believe that children should not play with children infected.

With regard to social cohesion/discohesion, feeling supported within the household (OR = 0.79 95% CI = 0.70–0.89), which was the case for only 7% of the respondents, increased the likelihood of being tested. Conversely, not getting along well with the spouse (OR = 1.19 95% CI = 1.06–1.35), not feeling supported by the spouse (OR = 1.14 95% CI = 1.02–1.27) and fear of being abandoned by the spouse (OR = 1.13 95% CI = 1.05–1.21) increased the odds of not being tested. Not getting along well with the neighbors (OR = 1.18 95% CI = 1.06–1.32), high levels of perceived stigma in the community (OR = 1.21 95% CI = 1.09–1.35) and fear of community gossip (OR = 1.12 95% CI = 1.05–1.19) also increased the risk of not being tested. In stratified analysis by location and gender, the fear of community gossip was positively associated with uptake of testing in women living in rural areas (OR = 0.56 95% CI = 0.32–0.99) while for men it remained a non-statistically significant risk (OR = 1.41 95% CI = 0.89–2.22). In urban areas the fear of community gossip was a risk factor for both women (OR = 1.52 95% CI = 1.02–2.27) and men (OR = 2.00 95% CI = 1.23–3.26). Being afraid of social rejection (OR = 1.25 95% CI = 1.16–1.35) also increased the odds of not being tested. The odd ratios reported in this section referred to a one unit increase in the respective 5-level score (score 1–5).

In Table 7 we display the results of the multivariable logistic regression model controlling for the random effect of location (PSUs). On the individual level being male, older (every five year increase in age), living in urban areas and having no education were all associated with non-uptake of testing. People married (OR = 0.57 95% CI = 0.37–0.88) had a lower risk of refusing the HIV test yet those who perceived a high tolerance of gender-based violence in their households (OR = 2.10 95% CI = 1.05–4.32) and did not get along well with the spouse (OR = 2.48 95% CI = 1.00–6.19) were twice more likely of not being tested. These effects were consistent with the unadjusted analysis and did not change when we conducted sensitivity analysis excluding from the analysis HIV positive participants who disclosed their status to a family member and who reported a change in the relationship in their couple after being tested. About 29% of married individuals reported conflicts within the couple of whom less than half (43.5%) were tested for HIV. Among couples who didn’t have marital conflicts the testing prevalence was about 74.1%. Similarly 20.2% of all married participants perceived a high tolerance to gender-based violence in their households. Furthermore being afraid of social rejection (OR = 1.48 95% CI = 1.23–1.80) also increased the odds of not being tested and this effect was strongly modified by the level of fear of community gossip. We tested the interaction between these two variables but it was not statistically significant. Further analysis suggested that community gossip would rather be a mediator of the association between fear of social rejection and uptake of HIV testing. To test this mediating effect, we ran a multinomial regression analysis using first the following categorical outcome: reference category (being tested), category 1 (being not tested but have no fear of community gossip) and category 2 (being not tested but have fear of community gossip). We computed the relative risk ratios (RRR) of each predictor associated with each outcome’s category and then computed the RRR of the contrast between outcome’s category 2 and 1 using the command LINCOM in Stata 12.1 (which works like changing the outcome’s reference category and running again the multinomial regression). The results of this analysis offered evidence in favor of our mediating effect hypothesis as the risk for not testing due to fear of social rejection (RRR = 3.44; 95%CI = 2.78–4.25) tripled when respondents were afraid of community gossip as compared to those who had no fear of gossip. This analysis also showed that high levels of community gossip doubled the risk that men were never tested. Alcohol abuse was also a risk factor for not being tested, even in the absence of community gossip. This was probably caused by the desire to avoid the stigma associated with a positive test result and be blamed for having contracted the virus because of drinking behavior. These results are presented in Table S1 in an online supplement.

**Table 7:** Adjusted odds ratios associated with non-uptake of HIV testing.

Risks determinants for not testing	AOR	P	95% CI
Urban*	2.31	**0.000**	1.45–3.69
Men*	1.64	**0.000**	1.34–2.02
Age*	1.12	**0.001**	1.05–1.19
No education*	2.00	**0.041**	1.03–3.88
Community participation	0.72	0.073	0.50–1.03
Married*	0.57	**0.012**	0.37–0.88
Respondent does not get along well with spouse/partner# *	2.48	**0.045**	1.00–6.19
Tolerance of gender based violence in the household# *	2.10	**0.041**	1.05–4.32
Alcohol abuse	1.12	0.093	0.98–1.27
Fear of social rejection score* 1	1.48	**0.000**	1.23–1.80
Fear of community gossip*[social control]	0.81	**0.014**	0.68–0.96
Random-effect: District/Village	0.43	0.186	0.18–1.01

* statistical significance p<0.05.

# reported per one unit increase in scale 1 to 5.

1 score included: Fear of divorce; Fear of losing friends; Fear of damaging the family reputation; Fear of not being able to get married; Fear of being rejected by sexual partners.

## Discussion

Our findings show that marital conflicts, individual perceptions of high tolerance to gender-based violence within the household and the fear of social ostracism put people at greater risk of not being tested. In addition fear of social rejection was a strong risk for not getting tested but only if people were also afraid of gossip in the community. All these fears and conflicts are a likely result of prevailing social norms in the community. Acceptance of these norms especially those related to marriage rules may promote gender power inequality which can lead to violence and social abuses. There is significant evidence that gender inequity and gender-based violence increase vulnerability to HIV infection [27], [28] and that gender-based violence and sexual risk may be linked through alcohol consumption [46]. Our study expands on this evidence by showing that not only enacted gender-based violence adds to the burden of HIV, but tolerance of gender-based violence within families per se jeopardizes uptake of HIV care. The provision of couple counseling within the intervention package for VCT programs offer an opportunity to address these problems but those that do not test will nevertheless not benefit from such efforts.

Fear of gossip (about oneself) in the community was the most reported reason to have not been tested for HIV. Sociological and psychological evidence has long established that gossip is not simply trivial chat but an efficient means of social control and moral instruction [47]–[54]. A recent study from South Africa showed that in communities with high HIV prevalence gossip is used to spread information considered relevant to the prevention of HIV/AIDS at the local level and also to instruct people about socially (un-) acceptable behaviors in the community. In the words of the author “*Gossip about AIDS does not only describe, but is prescriptive. It creates moral readings of behavior, linking AIDS to discourses of tradition, gender, and generational relationships*”. [55].

The problem of gossip further shows that community based strategies are urgently needed, in complement to individual and family-based interventions. Interventions research on how to influence social norms and mobilize community support and how to enforce the statutory family and criminal law are needed in order to mitigate the negative effects of marital conflicts on women in Zambia. The last report on Human Rights in Zambia (2007) concluded that the lack of enforcement and the culture of impunity for perpetrators of violence against women were key challenges for the country. This report argued that although the government had established special units to respond to violence against women, discriminatory attitudes within the system (police and judiciary) prevented women from reporting violence and that women were often pressured by law enforcement officials to withdraw the allegations of violence or for reconciliation with abusive husbands. [56].

Other than individual- and marriage-centered approaches such as couples counseling we suggest studying the potential value (capacity and authority) that traditional authorities of the clan/kinship system [57] or of the various churches could have for mobilizing the community and influencing negotiation within family networks on how marital conflicts and violence can be mitigated. The potentially effective leadership role of traditional leaders in Zambia for changing marital norms, such as to abolish informal marriage rules like levirate marriage (marriage with a brother’s widow or inheritance of the brother’s wife), has been previously highlighted [58] but less is known about the influence of religious leadership.

To our knowledge this is the first study providing quantitative evidence on the association between unequal power relations within couples, tolerance of gender-based violence within families and HIV care seeking behavior in Southern Africa. It is consistent with recent findings from Zambia, Zimbabwe and Kenya showing that a low tolerance for domestic violence is positively associated with greater acceptance of HIV testing among women although, in this study, the effect was statistically significant only in Kenya [59]. Our results also confirm those of other studies conducted in the United States [60], China [61] and recently in Zambia [62] reporting that family and couple relationships are instrumental in the prevention and treatment of HIV/AIDS, that gender-based abuses increases the risk of not getting tested [63] and that social and family capital have the potential to influence vulnerability to HIV in Sub-Saharan Africa [64], [65].

We are aware that the interpretation of our results is limited by the cross-sectional design of the study which does not allow establishing a causal relationship between couple conflicts, tolerance to gender based violence, fear of social rejection and uptake of HIV testing. However, the fact that we found similar associations in persons having disclosed their HIV-positivity or having reported a change in their relationship argues against inverse causality. We cannot rule out the possibility of hidden confounding factors that could explain these effects. However, in our models we consider a large set of variables that were selected based both on our previous knowledge of the topic and the context and on a comprehensive literature search. We further assessed whether the association between couple discohesion and HIV-testing was modified by age, sex and perceived stigma in the community but found no evidence of such interactions. Nonetheless longitudinal research is needed to clarify these potential causal relationships. Another limitation is that our analysis was based on self-reported prevalence of HIV testing. Yet, our results were similar to those of another study, which actually tested the respondents, [66] and was conducted in the same areas and during the same period. Thus we are confident that our data reflects the reality of the communities we studied. Finally, although our study had a limited geographical scope the sample in Lusaka was representative of a large urban environment in Zambia and, as the country has a high degree of urbanization, we assume that our results are nationally relevant. Likewise both rural areas and the peri-urban town that we surveyed offered three different rural environments increasing the likelihood that the study is generalizable to the Southern Zambian rural context as well.

## Conclusions

In Zambia, as in other parts of Sub-Saharan Africa, programmes to increase access to HIV care services have strongly relied on stigma reduction campaigns and the promotion of couple VCT. Although these interventions were correctly targeted and contributed to improve uptake [67]–[69] they did not aim to reduce power imbalances between men and women. Couple-testing may be most beneficial to couples with a mutually supportive relationship but it is unlikely that it adequately accommodates couples in conflict especially because, as our study shows, these couples have a higher risk of not being tested.

Even if services are enhanced and stigma is reduced prevailing gender inequality and tolerance of gender-based violence will continue to pose a significant barrier to uptake of testing unless there is real commitment to engage in social processes to reduce gender inequality. Not being able to freely decide whether to get tested due to fear of violence or social exclusion is a moral and human rights violation that can and must be urgently addressed. Programs to prevent AIDS and increase access to HIV care must be planned and designed using frames to protect and promote equal rights thus improving the participation of those who are most vulnerable. Addressing gender issues is not just a matter of including a focus on women and girls as a crosscutting issue in HIV/AIDS programming. Ending gender inequality requires political will and a comprehensive rights-based approach to HIV/AIDS.

## Supporting Information

Table S1 **Multinomial regression: community gossip as a mediator of the association between social rejection and community gossip.**
(DOCX)

## References

[1] UNAIDS (2009) Joint Action for Results. UNAIDS Outcome Framework 2009–2011.

[2] Gilbert L, El-Bassel N, Schilling RF, Wada T, Bennet B (2000) Partner violence and sexual HIV risk behaviors among women in methadone treatment. AIDS and Behav 4: 261–269.

[3] Decker MR, Seage GR, Hemenway D, Raj A, Saggurti N, et al (2009) Gender partner violence functions as both a risk marker and risk factor for women’s HIV infection: findings from Indian husband-wife dyads. J Acquir Immune Defic Syndr 51(5): 593–600. 19421070 10.1097/QAI.0b013e3181a255d6 PMC3521617

[4] Van der Straten A, King R, Grinstead O, Vittinghoff E, Serufilira A, et al (1998) Sexual coercion, physical violence, and HIV infection among women in steady relationships in Kigali, Rwanda. AIDS and Behavior 2(1): 61–73.

[5] UNAIDS (2010) Global Report Fact Sheet: Sub-Saharan Africa.

[6] UNAIDS (2009) AIDS Epidemic Update (2009) and Population Reference Bureau, The World’s Women and Girls (2011) Data Sheet (Washington DC: Population Reference Bureau, 2011).

[7] UNAIDS/WHO, Women and AIDS, An Extract from AIDS: Epidemic Update (December 2004). Available: http://data.unaids.org/gcwa/jc986-epiextract_en.pdf. Accessed 2011 Apr 4.

[8] Central Statistical Office (CSO), Ministry of Health (MOH), University of Zambia, and MEASURE Evaluation (2010) Zambia Sexual Behaviour Survey (2009) Lusaka, Zambia: CSO and MEASURE Evaluation.

[9] Fylkesnes K, Siziya S (2004) A randomized trial on acceptability of voluntary HIV counselling and testing. Tropical Medicine and International Health 9: 566–572. 15117300 10.1111/j.1365-3156.2004.01231.x

[10] Wolff B, Nyanzi B, Katongole G, Ssesanga D, Ruberantwari A, et al (2005) Evaluation of a home-based voluntary counselling and testing intervention in rural Uganda. Health Policy and Planning 20: 109–116. 15746219 10.1093/heapol/czi013

[11] Were W, Mermin J, Bunnell R, Ekwaru JP, Kaharuza F, et al (2003) Home-based model for HIV voluntary counselling and testing. The Lancet 361(9368): 1569. 10.1016/S0140-6736(03)13212-6 12737909

[12] Global HIV/AIDS response (2011) Epidemic update and health sector progress towards universal access: progress report.

[13] Hutchinson PL, Mahlalela X (2006) Utilization of voluntary counseling and testing services in the Eastern Cape, South Africa. AIDS Care. 18: 446–455. 10.1080/09540120500213511 16777636

[14] Weiser SD, Heisler M, Leiter K, Percy-de Korte F, Tlou S, et al (2006) Routine HIV testing in Botswana: A population-based study on attitudes, practices, and human rights concerns. PloS Medicine 3: e261. 16834458 10.1371/journal.pmed.0030261 PMC1502152

[15] Glick P, Sahn DE (2007) Changes in HIV/AIDS knowledge and testing behavior in Africa: how much and for whom?. J Popul Econ 20(2): 383–422.

[16] Wringe A, Isingo R, Urassa M, Maiseli G, Manyalla R, et al (2008) Uptake of HIV voluntary counselling and testing services in rural Tanzania: implications for effective HIV prevention and equitable access to treatment. Trop Med Int Health 13(3): 319–327. 18397395 10.1111/j.1365-3156.2008.02005.x

[17] Corbett EL, Dauya E, Matambo R, Cheung YB, Makamure B, et al (2006) Uptake of workplace HIV counseling and testing: A cluster-randomized trial in Zimbabwe. PLoS Medicine 4 3: e238. 10.1371/journal.pmed.0030238 PMC1483908 16796402

[18] Irungu TK, Varkey P, Cha S, Patterson JM (2008) HIV voluntary counseling and testing in Nakuru, Kenya: Findings from a community survey. HIV Medicine 9: 111–117. 18257773 10.1111/j.1468-1293.2007.00538.x

[19] Kakoko DC, Lugoe WL, Lie GT (2008) Voluntary testing for HIV among a sample of Tanzanian teachers: A search for socio-demographic and socio-psychological correlates. AIDS Care 18: 554–560. 10.1080/09540120500259779 16831782

[20] Pool R, Nyanzi S, Whitworth JA (2001) Attitudes to voluntary counselling and testing for HIV among pregnant women in rural south-west Uganda. AIDS Care 13(5): 605–615. 11571007 10.1080/09540120120063232

[21] Ekanem EE, Gbadegesin A (2004) Voluntary counseling and testing (VCT) for human immunodeficiency virus: A study on acceptability by Nigerian women attending antenatal clinics. African Journal of Reproductive Health 8: 91–100. 15623124

[22] Pettifor A, MacPhail C, Suchindran S, Delany-Moretlwe S (2010) Factors associated with HIV testing among public sector clinic attendees in Johannesburg, South Africa. Aids Behav 14(4): 913–921. 18931903 10.1007/s10461-008-9462-5

[23] Koku EF (2011) Desire for and uptake of HIV tests by Ghanaian women: The relevance of community level stigma. J Commun Health 36(2): 289–299. 10.1007/s10900-010-9310-1 20838860

[24] Kalichman SC, Simbayi LC (2003) HIV testing attitudes, AIDS stigma, and voluntary HIV counselling and testing in a black township in Cape Town, South Africa. Sex Transm Infect 79(6): 442–447. 14663117 10.1136/sti.79.6.442 PMC1744787

[25] Jürgensen M, Tuba M, Fylkesnes K, Blystad A (2012) The burden of knowing: balancing benefits and barriers in HIV testing decisions. A qualitative study from Zambia. BMC Health Serv Res 12: 2. 22222028 10.1186/1472-6963-12-2 PMC3268706

[26] Dunkle KL, Jewkes RK, Brown HC, Gray GE, Mcintyre JA, et al (2004) Gender-Based Violence, Relationship Power, and Risk of HIV Infection in Women Attending Antenatal Clinics in South Africa. Lancet 363(9419): 1415–1421. 15121402 10.1016/S0140-6736(04)16098-4

[27] Jewkes RK, Dunkle K, Nduna M, Shai N (2010) Gender partner violence, relationship gender power inequity, and incidence of HIV infection in young women in South Africa: a cohort study. The Lancet 367: 41–48. 10.1016/S0140-6736(10)60548-X 20557928

[28] Jewkes R, Sikweyiya Y, Morrell R, Dunkle K (2011) The Relationship between Gender Partner Violence, Rape and HIV amongst South African Men: A Cross-Sectional Study. PLoS One 6(9): e24256. 21935392 10.1371/journal.pone.0024256 PMC3173408

[29] UNIFEM, Violence against Women–Facts and Figures (2011) Available: www.unifem.org/gender_issues/violence_against_women/facts_figures.php.Accessed 2012 Nov 25.

[30] UNGASS (2011) Zambia Country Progress Report. March 2012.

[31] Kish L (1949) A procedure for objective respondent selection within the household. Journal of the American Statistical Association 44: 380–387.

[32] Central Statistical Office (CSO), Ministry of Health (MOH), University of Zambia, and MEASURE Evaluation (2010), Zambia Sexual Behaviour Survey (2009). Lusaka, Zambia: CSO and MEASURE Evaluation.

[33] Epstein NB, Baldwin LM, Bishop DS (1983) The McMaster Family Assessment Device. Journal of Marital and Family Therapy 9: 171–180.

[34] Molenaar IW, Sijtsma K (2000). MSP5 for Windows. A Program for Mokken Scale Analysis for Polytomous Items. Groningen: ProGamma.

[35] Hardouin JB (2004) MSP: Stata module to perform the Mokken Scale Procedure. Boston College Department of Economics.

[36] Loevinger J (1948) The Technique of Homogeneous Tests Compared with Some Aspects of Scale Analysis and Factor Analysis. Psychological Bulletin 45: 507–530. 18893224 10.1037/h0055827

[37] Molenaar IW, Sijtsma K (1984) Internal Consistency and Reliability in Mokkeńs Nonparametric Item Response Model. Tijdschr Onderwijsresearch 9(5): 257–268.

[38] Sampson RJ, Raudenbush SW, Earls F (1997) Neighborhoods and violent crime: a multilevel study of collective efficacy. Science 227: 918–924. 10.1126/science.277.5328.918 9252316

[39] Zou J, Yamanaka Y, Muze J, Watt M, Osterman J, et al (2009) Religion and HIV in Tanzania: influence of religious beliefs on HIV stigma, disclosure, and treatment attitudes. BMC Public Health 9: 75. 19261186 10.1186/1471-2458-9-75 PMC2656538

[40] Fox MP, Mazimba A, Seidenberg P, Crooks D, Sikateyo B (2010) Barriers to initiation of antiretroviral treatment in rural and urban areas of Zambia: a cross-sectional study of cost, stigma, and perceptions about ART. J Int AIDS Soc. 13: 8. 10.1186/1758-2652-13-8 PMC2847987 20205930

[41] Deacon H (2006) Towards a sustainable theory of health-related stigma: lessons from the HIV/AIDS literature. Journal of Community & Applied Social Psychology 16 (6): 418–425.

[42] Herek GM, Mitnick L, Burris S, Chesney M, Devine P, et al (1998) AIDS and stigma: a conceptual framework and research agenda. AIDS Public Policy J 13(1): 36–47. 10915271

[43] Genberg BL, Kawichai S, Chingono A, Sendah M, Chariyalertsak S, et al (2008) Assessing HIV/AIDS stigma and discrimination in developing countries. AIDS and Behavior 12(5): 772–780. 18080830 10.1007/s10461-007-9340-6 PMC2745205

[44] Holzemer WL, Uys LR, Chirwa ML, Greeff M, Makoae LN, et al (2007) Validation of the HIV/AIDS Stigma Instrument - PLWA (HASI-P). AIDS Care 19(8): 1002–12. 17851997 10.1080/09540120701245999

[45] Radimer KL, Olson CM, Campbell CC (1990) Development of indicators to assess hunger. J Nutr 120: 1544–1548. 2243303 10.1093/jn/120.suppl_11.1544

[46] Pitpitan EV, Kalichman SC, Eaton LA, Sikkema KJ, Watt MH, et al (2012) Gender-based violence and HIV sexual risk behavior: alcohol use and mental health problems as mediators among women in drinking venues, Cape Town. Social Science & Medicine 75 (8): 1417–1425. 10.1016/j.socscimed.2012.06.020 PMC3425436 22832324

[47] Andreassen R (1998) Gossip in Henningsvaer. Etnofoor 11(2): 41–56.

[48] Besnier N (1994) The truth and other irrelevant aspects of Nukulaelae gossip. Pacific Studies 17(3): 1–39.

[49] Sabini J, Silver M (1982) Moralities of Everyday Life. Oxford, UK: Oxford University Press.

[50] Hannerz U (1967) Gossip, networks and culture in a black American ghetto. Ethos 32: 35–60.

[51] Dunbar R (1992) Why gossip is good for you. New Scientist 136: 28–31.

[52] Cotts Watkins S, Swidler A (2009) Hearsay Ethnography: Conversational Journals as a Method for Studying Culture in Action. Poetics (Amst) 37(2): 162–184. 20161457 10.1016/j.poetic.2009.03.002 PMC2791322

[53] Gluckman M (1963) Gossip and scandal. Current Anthropology 4(3): 307–16.

[54] White L (2000) Speaking with vampires: Rumor and history in Colonial Africa. Berkeley CA: UCLA Press.

[55] Stadler J (2003) Rumour, gossip and blame: implications for HIV/AIDS prevention in the South African Lowveld. AIDS Education and Prevention 15(4): 357–368. 14516020 10.1521/aeap.15.5.357.23823

[56] OMCT (2007) Human Rights Violations in Zambia Part II: Women’s rights, Shadow Report: UN Human Rights Committee. Available: http://www.omct.org/files/2005/09/3065/zambia_omct_alt_report_hrc_women.pdf. Accessed 2013 Jan 11: p.9.

[57] Ankrah EM (1993) The impact of HIV/AIDS on the family and other significant relationships: the African clan revisited. Aids Care 5 (1): 5–22. 10.1080/09540129308258580 8461361

[58] Singh K, Luseno W, Haney E (2013) Gender equality and education: Increasing the uptake of HIV testing among married women in Kenya, Zambia and Zimbabwe AIDS Care. (Epub ahead of print). 10.1080/09540121.2013.774311 PMC3966089 23438082

[59] Malungo J (2001) Sexual Cleansing (Kusalaya) and Levirate Marriage (Kunjilila mung’anda) in the Era of AIDS: Changes in Perception and Practices in Zambia. Social Science and Medicine 53: 371–382. 11439820 10.1016/s0277-9536(00)00342-7

[60] Pequegnat W, Szapocznik J (2000) The role of families in preventing and adapting to HIV/AIDS: Issues and answers. In Pequegnat W, Szapocznik J. Working with families in the Era of HIV/AIDS (pp.3–26).Thousand Oaks CA: Sage Publications.

[61] Li L, Wu S, Wu Z, Sun S, Cui H, et al (2006) Understanding Family Support for People Living with HIV/AIDS in Yunnan, China. AIDS Behav 10(5): 509–517. 16741672 10.1007/s10461-006-9071-0 PMC2795327

[62] Vamos S, Cook R, Chitalu N, Mumbi M, Weiss SM, et al..(2013) Quality of relationship and sexual risk behaviors among HIV couples in Lusaka, Zambia. AIDS Care (Epub ahead of print). 10.1080/09540121.2012.749339 PMC3636183 23336258

[63] Adams JL, Hansen NB, Fox AM, Taylor BB, van Rensburg MJ, et al (2011) Correlates of HIV testing among abused women in South Africa. Violence against Women 17(8): 1014–23. 21727154 10.1177/1077801211414166 PMC3262407

[64] Poundstone KE, Strathdee SA, Celentano DD (2004) The social epidemiology of human immunodeficiency virus/acquired immunodeficiency syndrome. Epidemiologic Reviews 26: 22–35. 15234945 10.1093/epirev/mxh005

[65] Campbell C, Williams B, Gilgen D (2002) Is social capital a useful conceptual tool for exploring community level influences on HIV infection? An exploratory case study from South Africa. AIDS Care 14 (1): 41–54. 10.1080/09540120220097928 11798404

[66] Ayles HM, Schaap Ab, et al (unpublished) Results of the Zambia-South Africa TB and AIDS Reduction (ZAMSTAR) (2006–2009) Trials. 2008 Nov 7 9: 63 doi: 10.1186/1745-6215-9-63 10.1186/1745-6215-9-63 PMC2585552 18992133

[67] Farquhar C, Kiarie JN, Richardson BA, Kabura MN, John FN, et al (2004) Antenatal couple counseling increases uptake of interventions to prevent HIV-1 transmission. Journal of Acquired Immune Deficiency Syndromes 37: 1620–1626. 15577420 10.1097/00126334-200412150-00016 PMC3384734

[68] Conkling M, Shutes EL, Karita E, Chomba E, Tichacek A, et al (2010) Couples? Voluntary counselling and testing and nevirapine use in antenatal clinics in two African capitals: a prospective cohort study. J Int AIDS Soc 13: 10. 20230628 10.1186/1758-2652-13-10 PMC2851580

[69] Desgrées-du-Loû A, Orne-Gliemann J (2008) Couple-centred testing and counselling for HIV serodiscordant heterosexual couples in sub-Saharan Africa. Reproductive Health Matters 16(32): 151–161. 10.1016/S0968-8080(08)32407-0 19027631

