# Supplementary material for: HIV Testing and Tolerance to Gender Based Violence: A Cross-Sectional Study in Zambia
Source: PLoS One. 2013 Aug 21;8(8):e71922. doi: 10.1371/journal.pone.0071922 (PMC3749220; doi:10.1371/journal.pone.0071922)
Supplement: Table S1 — Multinomial regression: community gossip as a mediator of the association between social rejection and community gossip. (DOCX) [file pone.0071922.s001.docx]

# Table S1 Multinomial regression: community gossip as a mediator of the association between social rejection and community gossip

|  | **Category 1**  **Not tested & No fear of gossip *against* being tested** | | | **Category 2**  **Not tested & Fear Gossip**  ***against* being tested** | | | **Category 1 *against***  **Category 2** | | |
| --- | --- | --- | --- | --- | --- | --- | --- | --- | --- |
|  | **RRR** | **P>z** | **95% CI** | **RRR** | **P>z** | **95% CI** | **RRR** | **P>z** | **95% CI** |
| Urban | 2.12 | 0.006 | 1.24 - 3.64 | 3.25 | 0.000 | 1.81 - 5.85 | 1.54 | 0.240 | 0.75-3.14 |
| Men* | 1.73 | 0.009 | 0.38 - 2.60 | 4.65 | 0.000 | 2.57 - 8.41 | 2.69 | **0.002** | 1.46 - 4.97 |
| Age | 1.02 | 0.000 | 1.01 - 1.04 | 1.01 | 0.460 | 0.99 - 1.03 | 0.99 | 0.249 | 0.96-1.01 |
| No education | 2.41 | 0.011 | 1.22 - 4.77 | 0.45 | 0.468 | 0.05 - 3.83 | 0.19 | 0.104 | 0.25-1.41 |
| Community participation | 0.80 | 0.260 | 0.53 - 1.18 | 0.51 | 0.018 | 0.29 - 0.89 | 0.64 | 0.217 | 0.32-1.29 |
| Married | 0.55 | 0.027 | 0.32 - 0.93 | 0.66 | 0.195 | 0.35 - 1.24 | 1.20 | 0.673 | 0.51 - 2.81 |
| Alcohol abuse * | 1.19 | 0.012 | 1.04 - 1.36 | 0.95 | 0.624 | 0.79 - 1.15 | 0.80 | **0.035** | 0.65-0.98 |
| Couple's conflict_#_* | 1.84 | 0.290 | 0.59 – 5.37 | 3.05 | 0.098 | 0.81 - 11.50 | 1.69 | 0.530 | 0.32-8.38 |
| Domestic Violence_#_* | 2.19 | 0.019 | 1.16 – 4.16 | 1.76 | 0.284 | 0.62 - 5.00 | 0.82 | 0.733 | 0.25-2.59 |
| **Fear of social rejection score*_1_** | **0.77** | **0.006** | **0.63 - 0.93** | **2.77** | **0.000** | **2.29 - 3.35** | 3.44 | **0.000** | 2.78 - 4.25 |
| * statistical significance p<0.05  _#_ reported per one unit increase in scale 1 to 5  _1_ score included: Fear of divorce; Fear of losing friend ; Fear of damaging the family reputation; Fear of not being able to get married; Fear of being rejected by sexual partners | | | | | | | | | |
